# Interaction of prior category knowledge and novel statistical patterns during visual search for real-world objects

**DOI:** 10.1186/s41235-022-00356-y

**Published:** 2022-03-04

**Authors:** Austin Moon, Jiaying Zhao, Megan A. K. Peters, Rachel Wu

**Affiliations:** 1 Department of Psychology, University of California, 900 University Ave, Riverside, CA 92521 USA; 2 Department of Psychology and Institute for Resources, Environment and Sustainability, University of British Columbia, Vancouver, Canada; 3 Department of Cognitive Sciences, University of California, Irvine, USA; 4 Department of Bioengineering, University of California, Riverside, USA

## Abstract

Two aspects of real-world visual search are typically studied in parallel: category knowledge (e.g., searching for food) and visual patterns (e.g., predicting an upcoming street sign from prior street signs). Previous visual search studies have shown that prior category knowledge hinders search when targets and distractors are from the same category. Other studies have shown that task-irrelevant patterns of non-target objects can enhance search when targets appear in locations that previously contained these irrelevant patterns. Combining EEG (N2pc ERP component, a neural marker of target selection) and behavioral measures, the present study investigated how search efficiency is simultaneously affected by prior knowledge of real-world objects (food and toys) and irrelevant visual patterns (sequences of runic symbols) within the same paradigm. We did not observe behavioral differences between locating items in patterned versus random locations. However, the N2pc components emerged sooner when search items appeared in the patterned location, compared to the random location, with a stronger effect when search items were targets, as opposed to non-targets categorically related to the target. A multivariate pattern analysis revealed that neural responses during search trials in the same time window reflected where the visual patterns appeared. Our finding contributes to our understanding of how knowledge acquired prior to the search task (e.g., category knowledge) interacts with new content within the search task.

Efficient visual search for objects in the real world can be driven by prior knowledge about those objects (e.g., what they should look like, Maxfield et al., 2014; Robbins & Hout, 2020) and the current visual input (i.e., what the learner is currently seeing, Itti & Koch, 2001; Theeuwes, 2019). For example, in the real world, searching for a stop sign while driving can be facilitated by knowing that the sign is typically a red octagon with white letters (Olivers, 2011), as well as seeing several indications that a stop sign is approaching. How learners search for information based on their prior knowledge and how they process current visual input, especially in relation to visual patterns, have largely been studied independently.

Visual search studies have shown that when searching for objects from familiar categories (e.g., animate and inanimate objects, Cunningham & Wolfe, 2014; Drew et al., 2018), it can be challenging to ignore non-target objects that are conceptually related to the target (i.e., foils, Nako et al., 2014; Wu et al., 2015). In these studies, observers searched for multiple, related objects (i.e., objects within a category). While prior category knowledge facilitated search efficiency when searching for the whole category of objects (category search), it hindered efficiency when needing to search for specific objects and ignore related objects (foil effect). The foil effect can be measured using the N2pc component, the fastest and most robust ERP marker of attentional selection (Eimer, 1996; Luck & Hillyard, 1994). Prior studies have found that the foil N2pc suggests an involuntary activation of mental representations for a whole category (e.g., food), that contains the target (e.g., carrots, Wu et al., 2017). The N2pc is typically robust when finding a target and is attenuated, but often present, with foils.

Visual statistical learning studies have demonstrated the importance of visual patterns, such as for predicting future events (e.g., Baker et al., 2014; Luft et al., 2015; Wang et al., 2017; see Aslin & Newport, 2012 for a review). Visual statistical learning is largely an implicit process for extracting spatial and temporal patterns in the environment (e.g., Schapiro & Turk-Browne, 2015; Turk-Browne et al., 2005; Wang et al., 2019). It helps form the foundation of object identification and grouping, even from infancy (e.g., Fiser & Aslin, 2002; Kirkham et al., 2002; Wu et al., 2011). Interestingly, learners pay attention to visual patterns even when they are not relevant to the current task. For example, Zhao et al. (2013) found that reaction time was faster when targets appeared in the same location as a previous task-irrelevant visual pattern of nonsense symbols, compared to a location with a random stream of nonsense symbols.

The present study investigated how the use of prior knowledge (category knowledge of familiar objects) and novel information (statistical patterns with novel symbols) may interact during visual search, as measured via the N2pc ERP and behavioral measures. We predicted that target selection for objects in the same location as a previous visual pattern of symbols would yield larger N2pc components than in the location with a random sequence. We predicted that when foils appeared in lieu of a target, foil effects would emerge, but as attenuated N2pc components compared to target present trials, reflecting involuntary attention to items related to the target as a result of prior category knowledge. The Foil trials were designed to measure the influence of prior category knowledge, and we investigated the difference between the foil effects in the pattern versus random sequence locations. Although N2pc components typically appear 200–300 ms after the onset of targets and foils, the statistical patterns prior to target and foil onset may shift covert attention to these items sooner, because they are presented prior to the items. Therefore, to measure neural activity before the canonical N2pc time window, we investigated effects during a broader time window using signed negative area (150–300 ms, e.g., Sawaki et al., 2012). To further assess the differences in neural activity in this time window, we conducted a multivariate pattern analysis to decode neural responses (e.g., Bae & Luck, 2018, 2019; Bayet et al., 2018) based on stimulus location with respect to visual patterns. Onset latency was measured using a jackknife-based approach and fractional area latency to determine when shifts in covert attention occurred. Finally, we aimed to replicate the behavioral findings from Zhao et al. (2013) with real-world objects.

## Methods

### Participants

Nineteen adults (*M* = 19.32 years; *SD* = 1.86; range 18–25; 12 females; 7 males) were included in the final sample. Participants were recruited through a university system for course credit. One additional participant was excluded from the final analyses due to excessive eye movements (> 50% of accurate trials). The participants were 42% Asian, 16% Caucasian, 5% Native Hawaiian or other Pacific Islander, 5% mixed, 26% other, and 5% not specified. Seventeen out of 19 younger adults were right-handed. All participants had normal or corrected-to-normal vision. The experiment was approved by the IRB at UC Riverside and preregistered at AsPredicted: https://aspredicted.org/72yi5.pdf. We focused our analyses on the ERP and behavioral results and excluded other secondary preregistered variables (e.g., fixed vs. growth mindset).

### Stimuli

**Fig. 1:**
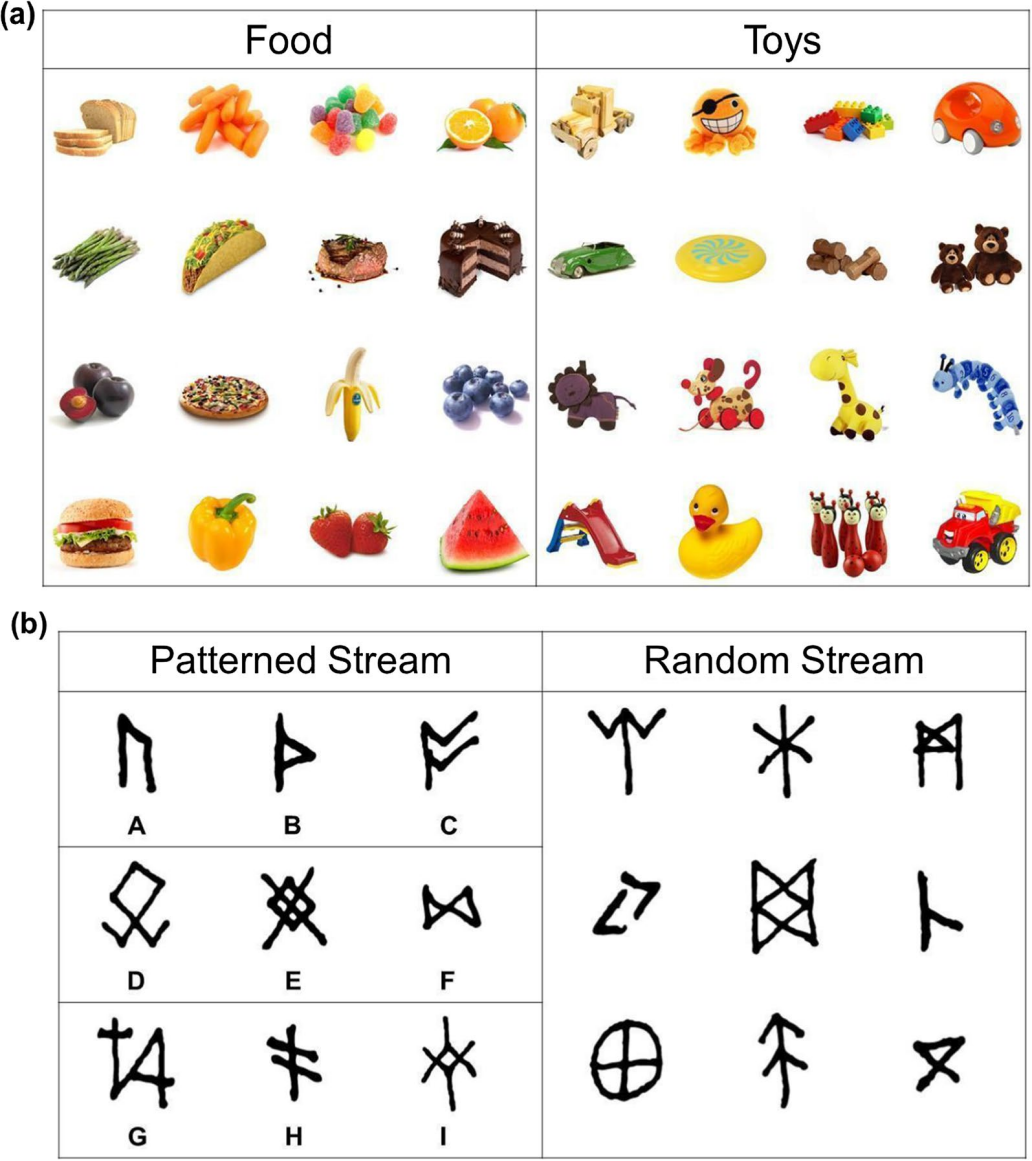
Search task stimuli. **a** Food and toy search objects. **b** Task-irrelevant patterned runic symbols (one set of three symbols make up a “triplet”: ABC, DEF, GHI) and randomly presented runic symbols

The real-world search stimuli consisted of 32 full-color real-world objects: 16 food objects (e.g., bread, banana) and 16 toy objects (e.g., rubber duck, Fig. 1a). These stimuli were visually matched based on overall shape and color. Task-irrelevant stimuli that were used for patterns consisted of 18 black runic symbols (Fig. 1b). All objects and symbols were presented on a white background using E-Prime 2.0 software. In the 2-object search array, all stimuli subtended 3.37° × 3.37° and were presented 3.13° left and right from the central fixation dot.

### Design

**Fig. 2:**
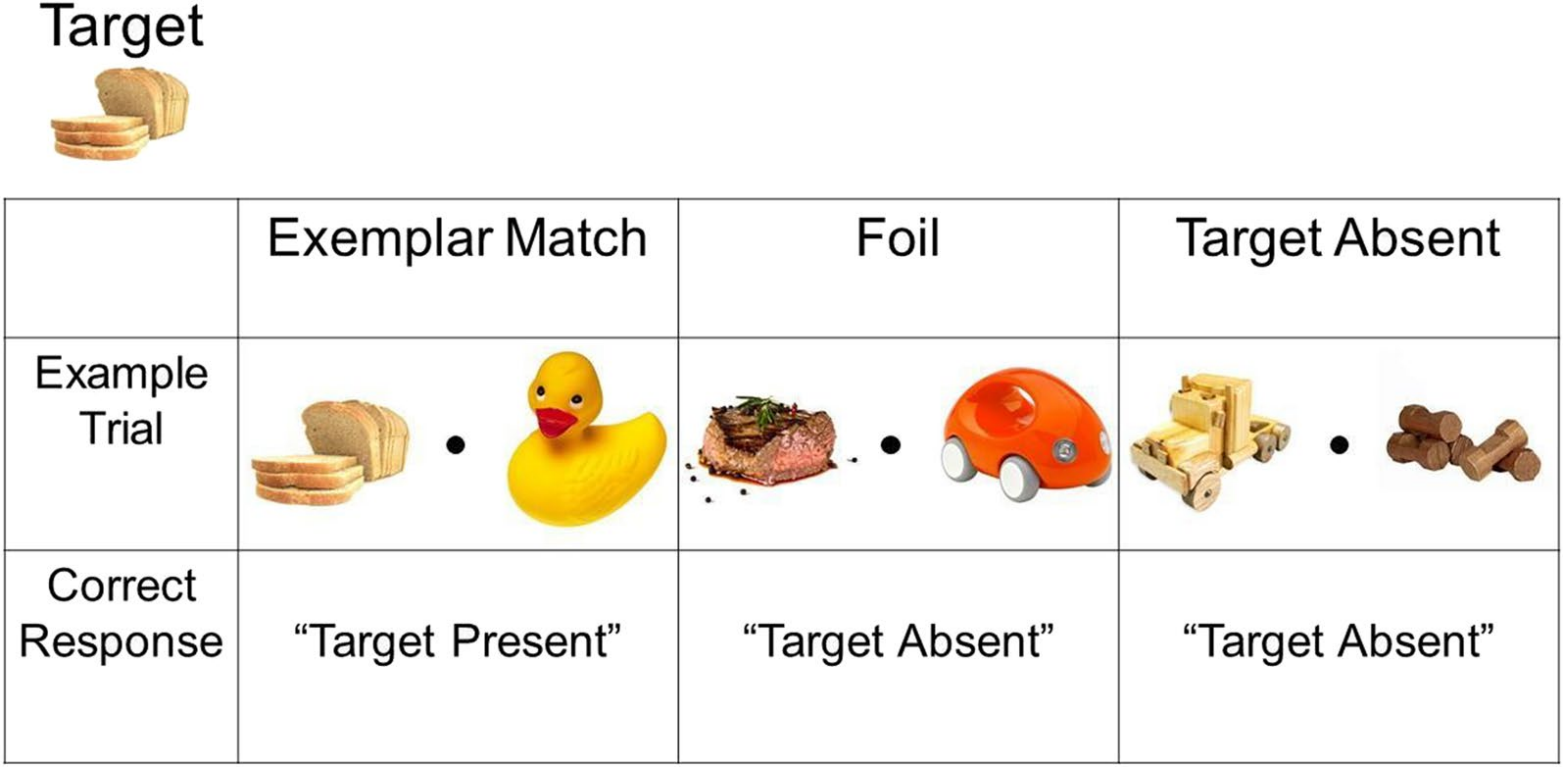
Sample trials for each trial type (Exemplar Match, Foil, and Target Absent trials) with bread as the target. Participants provided a binary response indicating whether the target (i.e., bread) was present (left arrow key) or absent (right arrow key). The target was held constant throughout the whole search task

Participants completed a search task for a specific item (e.g., search for bread; Fig. 2). Half of the participants searched for a food target, and the other half searched for a toy target. For each participant, the target was held constant for the entire search task. Each trial in the task contained a pattern phase and a search phase. The pattern phase included the presentation of the symbols in the two search locations: One location (either left or right of fixation dot) contained a symbol pattern, while the other contained a random pattern sequence. In the patterned sequence, nine symbols were grouped into three consistent triplets (e.g., ABC, DEF, GHI, but not ADG; Fig. 1b). In the random stream, runes were presented pseudo-randomly, with the restriction that back-to-back repeats were not allowed to avoid participants attributing these to some pattern. The pattern phase consisted of 1, 2, or 3 triplets (i.e., 3, 6, or 9 symbols). The location of the patterns was held constant throughout a search task for each participant and counterbalanced across participants.

The search phase followed the pattern phase and depicted two objects (food or toys) in the same two locations as the symbols. In contrast to Zhao et al. (2013), the search phase in the present study always followed the pattern phase after the presentation of the triplets had completed (i.e., with 3, 6, or 9 symbols). In addition, while Zhao et al. (2013) displayed four images during each trial with letters as the targets and distractors (i.e., L’s vs. T’s), our paradigm presented two images per trial with real-world images (i.e., food or toys). The types of objects that appeared varied according to the three trial types: (1) Exemplar Match trials, where the target object (e.g., bread) was present in one location, while an object from the other category (e.g., rubber duck) was present in the opposite location; (2) Foil trials, where a non-target object from the target’s category (e.g., steak) appeared in one location, while an object from the other category was present in the opposite location; and (3) Target Absent trials, where no objects from the target’s category (e.g., food) were presented, and only objects from the other category (e.g., toys) were presented, with the restriction that no two same non-targets were displayed simultaneously. Zhao et al. (2013) did not include Foil trials nor Target Absent trials.

In total, participants completed seven blocks (60 trials per block, 2 phases per trial), with a few exceptions. Each block consisted of 28 Exemplar Match trials, 28 Foil trials, and four Target Absent trials. The Exemplar Match trials displayed 14 trials with the target (e.g., bread) on the left and 14 trials with the target on the right. The Foil trials also displayed 14 trials with the foil (e.g., steak) on the left and 14 trials on the right. All 60 trials were randomized per block. The foils and non-target objects from the opposing category (e.g., 16 toy objects if the target was a food object) were randomly selected during each trial.

### Procedure

**Fig. 3:**
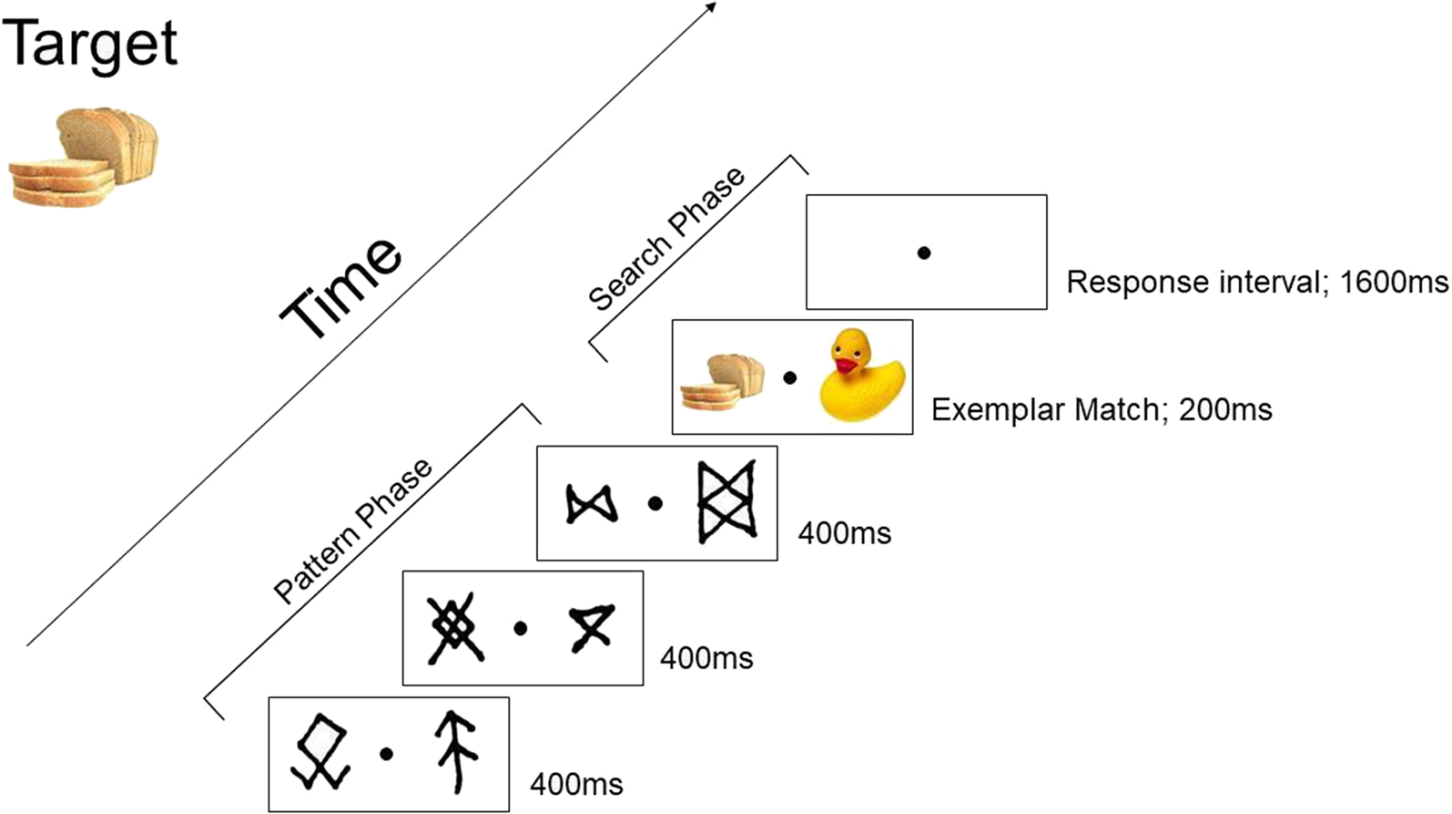
Sample trial sequence of the search task with bread as the target, which was held constant throughout the search task. The pattern phase could include 1 triplet (as pictured), or 2 or 3 triplets

At the start of the search task, participants were instructed to ignore the irrelevant runes because they were “spacers” for the search task. This comment helped ensure that participants thought of these symbols as task-irrelevant. The exact target was indicated on the screen prior to the task and held constant throughout the whole task. During the search phase, participants indicated the presence of a target by pressing the left arrow key or the absence of a target by pressing the right arrow key with their right hand. Each symbol was displayed on the screen for 400 ms. Therefore, three triplets (9 consecutive symbols) would last 3600 ms. For the search phase, the objects appeared on the screen for 200 ms, followed by a 1600 ms response interval, which displayed a blank screen with only a fixation dot. Participants had to respond within this 1600 ms period indicating the presence or absence of the target (Fig. 3). After the visual search task, participants completed a two-alternative forced recognition task, where they were shown 60 trials of three simultaneously presented symbols in the upper half and three symbols in the lower half of the screen. One set of symbols was a true triplet, while the other set was a random triplet. Participants were asked to choose which of the two grouped symbols seemed like they belonged together and rated how confident they were in their responses (on a scale from 1 to 4). Afterward, participants were asked if they noticed anything strange about the task and if they used any strategies to maximize their performance.

### EEG recording and data reduction

EEG was DC-recorded at 500 Hz from 32 scalp electrodes using the extended 10/20 system with the Brain Products system. We applied a 40 Hz Butterworth zero phase IIR low-pass filter (48 dB/octave), a 0.1 Hz high-pass filter (12 dB/octave), and a 60 Hz notch filter after re-referencing the EEG to averaged earlobes. Baseline correction was applied − 100 to 0 ms prior to stimulus onset. Epochs were created from − 100 ms to 500 ms relative to stimulus onset. We applied the following artifact rejection criteria: horizontal EOG exceeding ± 25 μV (0 ms to 300 ms), vertical EOG exceeding ± 60 μV(0 ms to 300 ms), and all other channels exceeding ± 80 μV (− 100 ms to 500 ms). Only correct trials were included in the final ERP analyses. Target Absent trials were excluded from the EEG analyses because they did not contain a reference object and were excluded from the behavioral data. A time window of 200–300 ms was applied to measure mean N2pc amplitude at lateral posterior electrodes PO7 and PO8. Grand average waveforms were calculated from the ipsilateral and contralateral electrodes, with respect to the target and the foil when they appeared in the patterned and random locations. Averaged across participants, 95% (*M* = 260 trials, *SD* = 69) of all correct trials were retained on average after eye-movement artifact rejection.

## Results

### N2pc: 200–300 ms after stimulus onset (planned analyses)

**Fig. 4:**
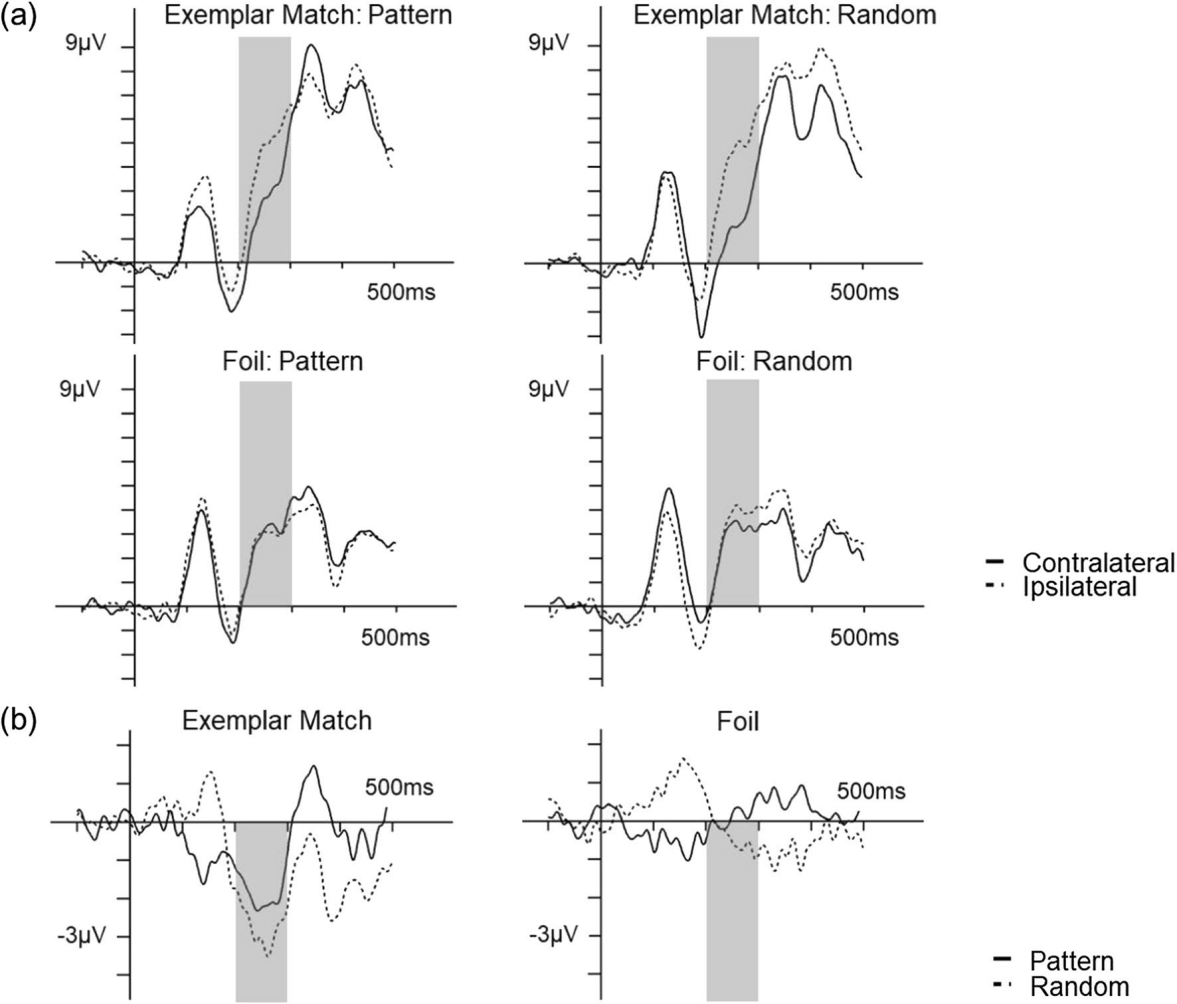
**a** Grand average contralateral and ipsilateral waveforms at electrodes PO7 and PO8 for Exemplar Match and Foil trials (with respect to the locations of the target or foil), separated by locations that contained patterned and random symbols. **b** Difference waveforms were calculated by subtracting the contralateral and ipsilateral waveforms

**Fig. 5:**
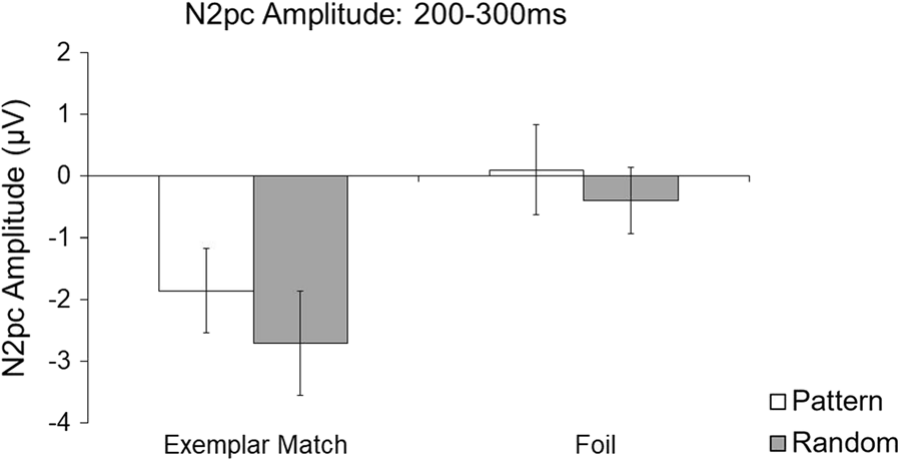
The mean amplitudes from the difference waveforms in the 200–300 ms (N2pc component) time windows. Error bars represent ± 1 SE. *p < .05

*Presence of the N2pc* The first analysis investigated the presence of the N2pc component when the targets and foils appeared in the patterned or random locations from 200 to 300 ms after stimulus onset (Figs. 4 and 5). One-sample *t* tests of mean N2pc amplitude (compared to 0 μV) revealed a significant N2pc during the Exemplar Match trials, when the targets appeared in the patterned location (*M* = − 1.85 μV, *SD* = 3.01), *t*(18) = − 2.68, *p* = 0.015, *d* = − 0.61, and in the random location (*M* = − 2.71 μV, *SD* = 3.69), *t*(18) =  − 3.20, *p* = 0.005, *d* = − 0.74. There was no significant N2pc when foils appeared in the patterned location (*M* = 0.10 μV, *SD* = 3.18), *t*(18) = 0.14, *p* = 0.891, *d* = 0.03, or in the random location (*M* = − 0.40 μV, *SD* = 2.36), *t*(18) = − 0.73, *p* = 0.474, *d* = − 0.17.

*Omnibus ANOVA* The next analysis investigated the effects of symbol position (patterned versus random) and trial types (Exemplar Match and Foil trials) on the mean amplitude. A 2 (symbol position: pattern and random) × 2 (trial type: Exemplar Match and Foil trials) ANOVA revealed a main effect of trial type, *F*(1,18) = 21.31, *p* < 0.001, η^2^_p_ = 0.54, where Exemplar Match trials (*M* = − 2.28 μV, *SD* = 1.74) had a larger mean N2pc amplitude than Foil trials (*M* = − 0.15 μV, *SD* = 1.02). There was no main effect of symbol position, *F*(1,18) = 0.32, *p* = 0.581, η^2^_p_ = 0.02, nor an interaction, *F*(1,18) = 0.25, *p* = 0.621, η^2^_p_ = 0.01.

Mean amplitudes at the standard time window showed a significant N2pc component when targets appeared in both the patterned and random locations. There were no significant N2pc components when foils appeared in either location, suggesting possibly that foil effects did not occur, irrespective of where foils appeared. Although N2pc components emerged sometime in the 200–300 ms time window for Exemplar Match trials, there were no differences when the target appeared in the patterned nor random location. However, it is possible that visual patterns shifted covert attention sooner than 200 ms. To best select an optimal time window that captures N2pc components for all participants while minimizing noise, signed negative area (Gaspar & McDonald, 2018; Gaspar et al., 2016; Sawaki et al., 2012; Tay et al., 2019) was measured at a broad time window of 150–300 ms.

**Fig. 6:**
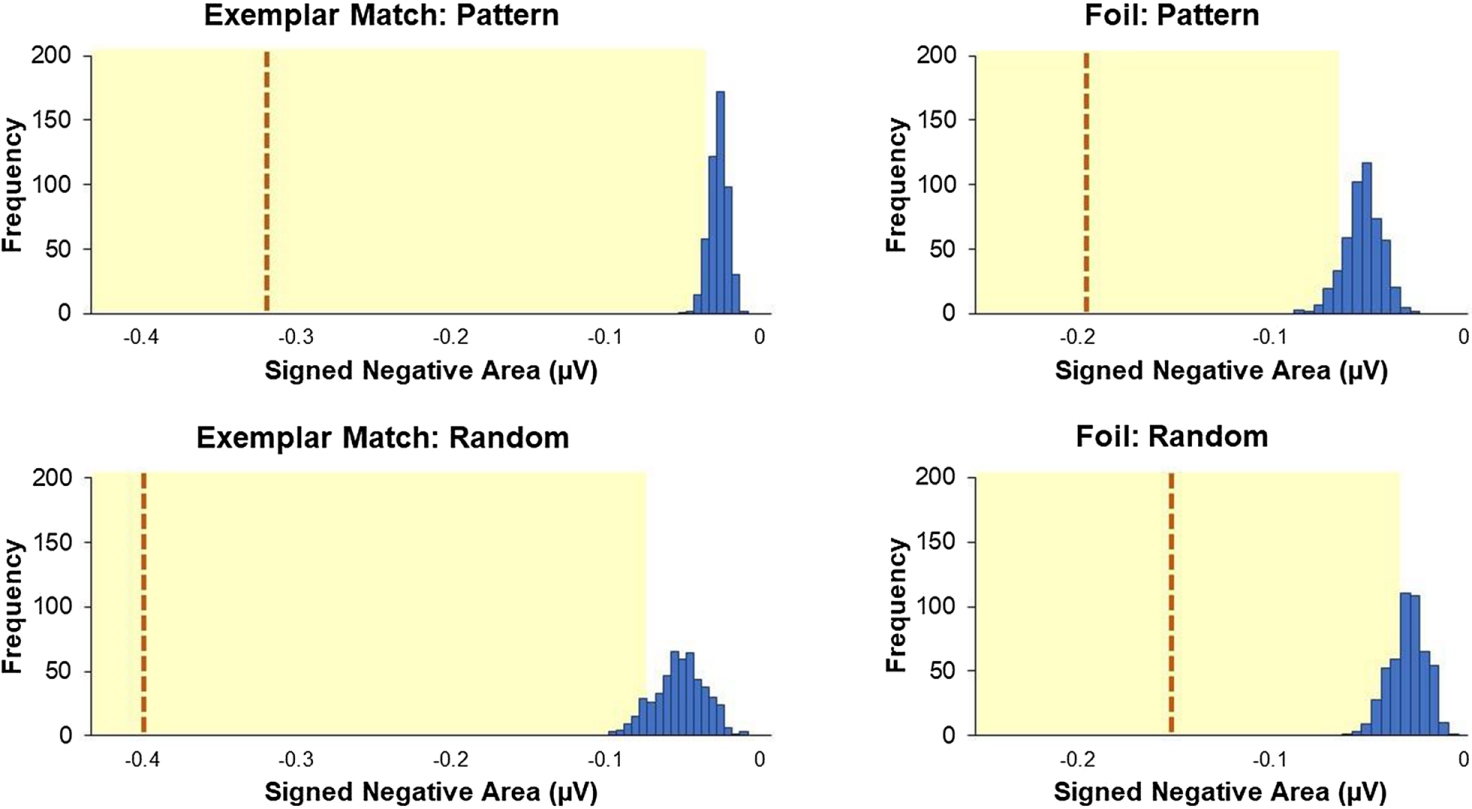
Permutation tests of the signed negative areas within the 150–300 ms time window after stimulus onset. Vertical blue bars represent distributions of areas from grand averages without signal (null distribution). Shaded yellow regions indicate areas above the 95th percentile of the permuted grand average values. The vertical orange dashed lines indicate the observed area from the original dataset

*Signed negative area* Signed negative areas were used to compute the magnitude of the N2pc component across a broad time window of 150–300 ms. Due to variability in noise with areas and bias to nonzero values, a nonparametric permutation test (e.g., Sawaki et al., 2012; Tay et al., 2019) was used in lieu of traditional one-sample *t* tests compared to zero. The dataset was permuted to create a distribution of expected values assuming the null hypothesis is true. Specifically, for each participant, individual trials were randomly assigned one of two possible conditions (patterned vs. random location), separately for Exemplar Match and Foil trials, to estimate noise while subtracting signal. The random assignment process occurred 500 times, and each time, a grand average of the four conditions (2 symbol positions × 2 trial types) was calculated. For each grand average, a signed negative area was obtained, with a total of 500 areas for each condition. Figure 6 shows the null distribution of the 500 signed negative areas for each condition. The *p* value was based on the proportion of times the observed area (i.e., area from grand average with correct assignment of conditions) was larger (in this case, more negative) than the permuted value (see Eq. 1 in Tay et al., 2019). The *p* value was considered significant when the observed value exceeded the 95th percentile of the null distribution.

For Exemplar Match trials, the signed negative area was significant when targets appeared in the patterned location (*M* = 0.32 μV, *SD* = 0.34), *p* = 0.002, and in the random location (*M* = 0.41 μV, *SD* = 0.35), *p* = 0.002. Similar results were found with the Foil trials, in the patterned location (*M* = 0.20 μV, *SD* = 0.26), *p* = 0.002, and in the random location (*M* = 0.15 μV, *SD* = 0.15), *p* = 0.002. Given that the effects for all conditions were well beyond what was to be expected under the null (i.e., top 5% values), it is unlikely that the effects were due to chance. A 2 (symbol position) × 2 (trial type) ANOVA revealed no main effect of symbol position, *F*(1,18) = 0.04, *p* = 0.849, η^2^_p_ = 0.20. There was a main effect of trial type, *F*(1,18) = 24.58, *p* < 0.001, η^2^_p_ = 0.58, with a larger N2pc area for Exemplar Match trials compared to Foil trials. There was also an interaction between symbol position and trial type, *F*(1,18) = 4.52, *p* < 0.048, η^2^_p_ = 0.20. Pairwise comparisons did not reveal a difference between patterned and random location for either Exemplar Match trials, *t*(18) =  − 0.67, *p* < 0.51, or Foil trials, *t*(18) = 0.54, *p* < 0.60.

*Multivariate pattern analysis (MVPA)* To examine whether scalp distributions were sensitive to spatial information (i.e., locations that contained visual patterns), an exploratory MVPA with support vector machines (SVMs) was implemented in MATLAB 2021a, with the EEGLAB (Delorme & Makeig, 2004) and ERPLAB (Lopez-Calderon & Luck, 2014) toolbox. The decoding procedures were adapted from Bae and Luck (2018, 2019) and were run separately for Exemplar Match and Foil trials. Briefly, for each participant’s preprocessed data, SVMs were trained to distinguish scalp responses (excluding HEOG and reference electrodes) at each time point when items appeared in the patterned location vs. random location. A threefold cross-validation (10 iterations) was used, where the data were randomly divided into three separate blocks with an equal number of trials. For each block, the trials were averaged to increase signal-to-noise ratio. For each iteration, two of the three blocks were randomly selected for training, and the last block was used for testing. Decoding accuracy was based on comparing the true label (i.e., patterned or random location) with the predicted label, with a chance performance of 0.50 (= 1/2). For each participant, decoding accuracy was averaged as a proportion across 60 decoding attempts (2 symbol positions × 3 cross-validations × 10 iterations) for each time point (60 for each trial type).

*Decoding analysis* First, the decoding performance was averaged across the broad time window of 150–300 ms (i.e., the likely window in which shift in covert attention occurred), and then compared to chance performance (0.50). A one-sample *t* test revealed decoding accuracy was well-above chance for both Exemplar Match trials (*M* = 0.71, *SD* = 0.14), *t*(18) = 6.35, *p* < 0.001, *d* = 1.46, and Foil trials (*M* = 0.55, *SD* = 0.06), *t*(18) = 3.19, *p* = 0.005, *d* = 0.73. To examine the differences in decoding performance between the trial types, a pairwise comparison revealed better performance for Exemplar Match trials than for Foil trials, *t*(18) = 4.13, *p* < 0.001, *d* = 0.95.

**Fig. 7:**
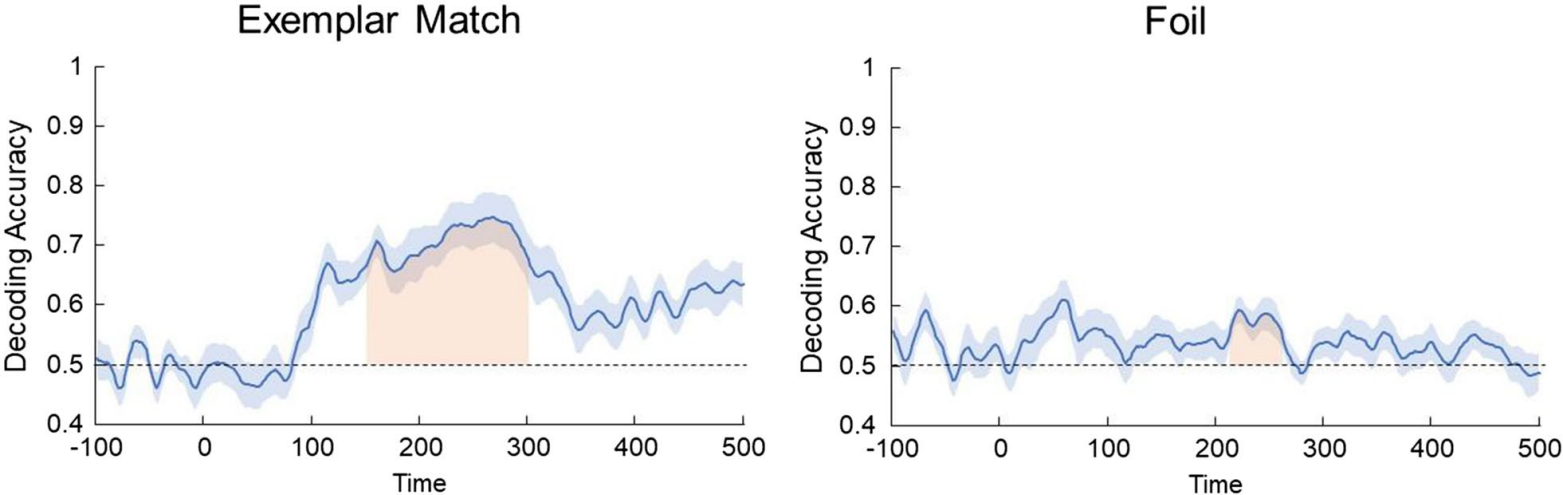
Average accuracy for decoding the symbol position with Exemplar Match (left) and Foil (right) trials. For each trial type, trials were assigned based on where items appeared (i.e., patterned vs. random location). Horizontal dashed lines represent chance performance (= 0.50). Red regions represent clusters of time points with significantly above-chance performance after correction. Error bars (blue shade) represent ± 1 SE

Second, a one-sample *t* test compared to chance was computed for each time point in the 150–300 ms time window. Then, clusters of contiguous significant time points were used to compute cluster-level *t* mass (i.e., the sum of *t* scores within a cluster). To control for Type I error, a null distribution of cluster-level *t* mass values was created based on permutation tests (see Bae & Luck, 2018). A permutation test iterated 10,000 times (a total of 10,000 *t* mass values assuming the null), and the *p* value of the observed *t* mass was considered significant if the observed *t* mass exceeded the top 95% of the null distribution (i.e., *p* < 10^–4^). For Exemplar Match trials, there was a significant cluster (*p* < 0.0001, one-tailed) across the whole 150–300 ms time window (Fig. 7), compared to the null distribution (critical *t* mass = 31.25, α = 0.05). For Foil trials, there was a significant cluster (*p* < 0.0001, one-tailed) between 212 and 262 ms, compared to the null (critical *t* mass = 26.29, α = 0.05).

#### Onset latency of N2pc

*Jackknife latency* A jackknife latency analysis was conducted using a − 0.75 µV threshold (Kiesel et al., 2008) for N2pc studies (methods described in Miller et al., 1998). Eighteen out of 19 participants’ data met the threshold for all four conditions (= 2 symbol positions × 2 trial types). The last participant’s data met the threshold for Exemplar Match trials in both the patterned and random location but only Foil trials in the patterned location. Therefore, only the data from Exemplar Match trials were included in the pairwise comparisons. A 2 (symbol position) × 2 (trial type) ANOVA revealed a main effect of symbol position, *F*(1,17) = 759.87, *p* < 0.001, η^2^_p_ = 0.98, with a faster latency when items appeared in the patterned location (*M* = 137.42 ms, *SD* = 3.88) than in the random location (*M* = 226.39 ms, *SD* = 10.96). There was a main effect of trial type, *F*(1,17) = 491.49, *p* < 0.001, η^2^_p_ = 0.97, with a faster latency for Exemplar Match trials (*M* = 156.21 ms, *SD* = 2.51) than for Foil trials (*M* = 207.67 ms, *SD* = 9.20). There was an interaction between symbol position and trial type, *F*(1,17) = 77.09, *p* < 0.001, η^2^_p_ = 0.82. A pairwise comparison revealed a shorter latency when targets appeared in the patterned location (*M* = 124.42 ms, *SD* = 4.93) than in the random location (*M* = 188.00 ms, *SD* = 2.31), *t*(18) = − 47.50, *p* < 0.001, *d* = − 10.90. Similarly, latency was shorter when foils appeared in the patterned location (*M* = 150.42 ms, *SD* = 4.60) than in the random location (*M* = 265.00 ms, *SD* = 21.67), *t*(17) =  − 19.15, *p* < 0.001, *d* = − 4.51.

*Fractional area latency* A fractional area latency analysis was conducted to detect potential early onset of N2pc components prior to the standard time window of 200–300 ms. Based on onset latencies with the jackknife approach, we chose a broad time window of 150–300 ms with a 50% negative area. Fifteen out of the 19 participants’ data met the threshold for all four conditions (= 2 symbol positions × 2 trial types). Three other participants’ data met the threshold for Exemplar Match trials in both patterned and random location but only Foil trials in the patterned location. Therefore, only the data with Exemplar Match trials were included in the pairwise comparisons. The last participant’s data met the threshold for both Exemplar Match and Foil trials in the patterned location but not in the random location. Therefore, these data were not included in any analyses. A 2 (symbol position) × 2 (trial type) ANOVA revealed no main effect of symbol position, *F*(1,14) = 2.58, *p* = 0.131, η^2^_p_ = 0.16, nor a main effect of trial type, *F*(1,14) = 2.31, *p* = 0.151, η^2^_p_ = 0.14. There was an interaction between symbol position and trial type, *F*(1,14) = 7.88, *p* = 0.014, η^2^_p_ = 0.36. A pairwise comparison revealed no difference in latencies between the patterned (*M* = 238.11 ms, *SD* = 26.55) and random (*M* = 240.22, *SD* = 28.75) locations for Exemplar Match trials, *t*(17) = − 0.23, *p* = 0.82. However, latencies were shorter when foils appeared in the patterned location (pattern: *M* = 210.80, *SD* = 42.51), than in the random location (random: *M* = 248.00, *SD* = 30.47), *t*(14) = − 2.23, *p* = 0.042, *d* = − 0.58.

*Data quality of ERP measures using standardized measurement error* To further test the data quality of ERP measures for the N2pc component, the bootstrapped standardized measurement error (*bSME*; see Luck et al., 2019) was calculated for the fractional area latency, mean amplitude, and signed negative area. Briefly, for each participant’s data, a simulation of the experiment was conducted 10,000 times, per condition, by sampling randomly with replacement from the correct trials after artifact rejection. For each iteration, the averaged waveform was made, and the three measures (i.e., fractional area latency, mean amplitude, and signed negative area) were computed, with a total of 10,000 values each. After completing the iterations, the *bSME* is calculated, which is the standard deviation of all 10,000 values. In total, each participant had 12 *bSME* values (2 symbol positions × 2 trial types × 3 measures). To get the average data quality across participants, we calculated the root mean square (*RMS*), or the aggregate of *bSME*’s of all participants, resulting in 12 *RMS* values (2 symbol positions × 2 trial types × 3 measures).

Currently, there is no conventional method to determine an ideal threshold for a “good” *RMS* value, let alone specific for N2pc components. However, one preliminary approach is to compare the *RMS* to the standard deviation of the group mean (from the observed grand average waveform), which is influenced by both true differences between participants and measurement error. A lower *RMS* value likely indicates that the observed variability was driven by true differences, instead of measurement error. For fractional area latency, the *RMS* values were the following: Exemplar Match with patterned location, 17.37 (compared to *SD* = 26.55); Exemplar Match with random location, 25.59 (compared to *SD* = 28.75); Foil with patterned location, 14.09 (compared to *SD* = 42.51); Foil with random location, 14.42 (compared to *SD* = 30.47). For mean amplitude, the *RMS* values were the following: Exemplar Match with patterned location, 0.82 (compared to *SD* = 3.01); Exemplar Match with random location, 0.83 (compared to *SD* = 3.69); Foil with patterned location, 0.78 (compared to *SD* = 3.18); Foil with random location, 0.76 (compared to *SD* = 2.36). For the signed negative area, the *RMS* values were the following: Exemplar Match with patterned location, 0.08 (compared to *SD* = 0.29); Exemplar Match with random location, 0.07 (compared to *SD* = 0.24); Foil with patterned location, 0.08 (compared to *SD* = 0.21); Foil with random location, 0.08 (compared to *SD* = 0.11). For all measures and conditions, the *RMS* values were moderately or much lower than the sample standard deviation, which suggests strong precision (i.e., low noise level) in the ERP measurements.

**Fig. 8:**
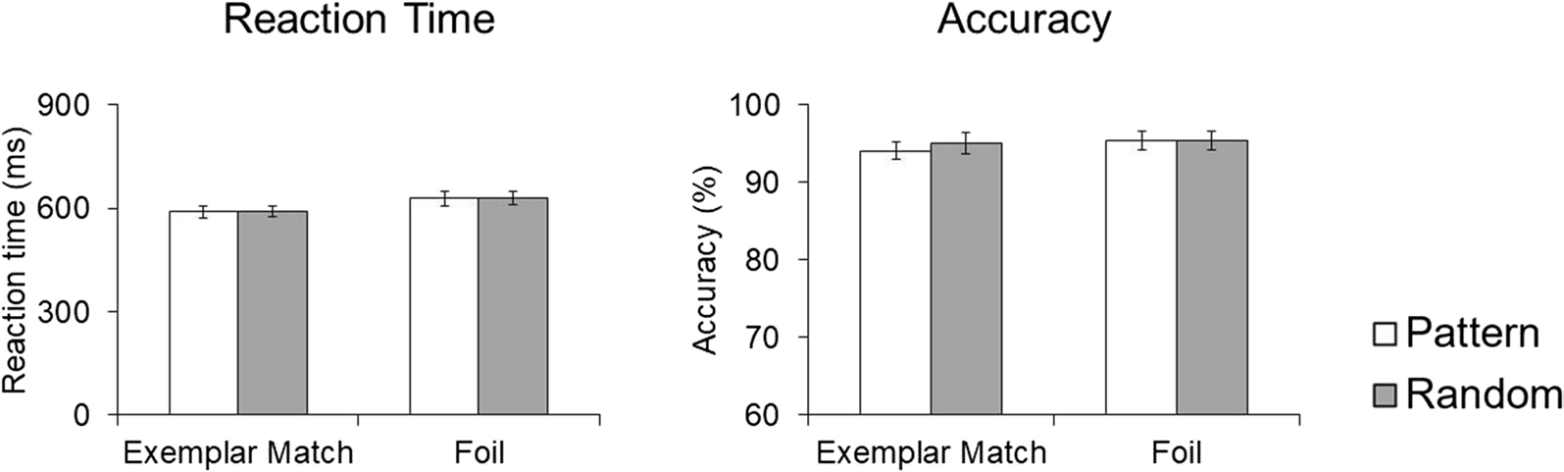
Reaction time and accuracy for Exemplar Match and Foil trials. Error bars represent ± 1 SE

*Behavioral results* To measure the effects of the symbol position and trial types on mean reaction time (RT) for correct trials, a 2 (symbol position) × 2 (trial type) ANOVA revealed a main effect of trial type, *F*(1,18) = 27.03, *p* < 0.001, η^2^_p_ = 0.60, with a faster RT for Exemplar Match trials (*M* = 616.21 ms, *SD* = 81.40) than for Foil trials (*M* = 660.14 ms, *SD* = 92.30). There was no main effect of symbol position, *F*(1,18) < 0.35, *p* = 0.563, η^2^_p_ = 0.02, nor an interaction between symbol position and trial type, *F*(1,18) < 0.18, *p* = 0.678, η^2^_p_ = 0.01 (Fig. 8). To measure the same effects as RT on accuracy, a 2 (symbol position) × 2 (trial type) ANOVA revealed no main effect of trial type, *F*(1,18) = 0.96, *p* = 0.340, η^2^_p_ = 0.0.05, no main effect of symbol position, *F*(1,18) = 0.39, *p* = 0.540, η^2^_p_ = 0.02, and no interaction between trial type and symbol position, *F*(1,18) = 0.44, *p* = 0.514, η^2^_p_ = 0.024.

*2AFC results* The two-alternative forced choice recognition task did not reveal an explicit preference for triplets (i.e., runes in the patterned location) over non-triplets (i.e., runes in the random location), *t*(18) = 0.66, *p* = 0.517, *d* = 0.15 (*M* = 0.51%, *SD* = 0.05). The participants provided an average confidence rating (scale from 1 to 4, 4 being highest confidence) of *M* = 2.74, *SD* = 0.28. In addition, participants did not explicitly report any suspicion of the symbols, nor did they report implementing any strategies during the search task.

## Discussion

Given that visual patterns and category knowledge can guide search in the natural environment, the present study examined the neural and behavioral responses when doing so. Prior behavioral findings have shown that performance was better when selected targets were in locations that previously included a patterned visual sequence, compared to when selected targets were in locations that previously included only a random sequence (Zhao et al., 2013). Based on this behavioral finding, we predicted that the size of the N2pc amplitude would follow the behavioral finding (i.e., larger for targets in patterned locations compared to random locations). Although we found a robust N2pc amplitude when targets appeared in either symbol positions during the standard time window of 200 ms to 300 ms after stimulus onset, the amplitudes did not differ between the two locations. These null N2pc results mirrored the null behavioral results. However, if there was an initial shift in attention to visual patterns, then it is possible that the N2pc component can be observed sooner than 200 ms. To investigate potential early neural effects, we found that the signed negative areas starting from 150 ms revealed N2pc components when targets and foils appeared in either location. Second, decoding accuracy revealed that symbol positions during task-relevant trials can be distinguished for both targets and foils, suggesting that scalp responses contained information about where the visual patterns appeared. The time window where symbol position can be discerned overlaps with the 150–300 ms ERP time window, which may be related to shifts in covert attention. Third, the latency results overall show shorter onset of the N2pc component for the patterned location compared to the random location. However, only the jackknife approach showed shorter onset with both targets and foils in favor of the patterned location, whereas the fractional area latency showed faster onset with foils only. Finally, the null results from the two-alternative forced recognition task suggest that participants did not have explicit knowledge about the patterns, which aligned with their lack of awareness and lack of search strategies reported during debriefing.

The N2pc components were based on two measures: mean amplitude during the standard 200–300 ms time window and signed negative area within the exploratory 150–300 ms time window. Although both measures revealed N2pc components for target items, only the negative areas revealed foil effects. One possible reason why the mean amplitude did not show a foil effect is that the standard time window was too narrow to capture N2pc components, as shown with latencies for foils in the patterned location (i.e., prior to 200 ms). It is unclear why mean amplitude did not show a significant foil effect for the random location, where latency was within the standard window (~ 250 ms). However, signed negative areas, which revealed foil effects, allow for selecting consistent time windows that contain N2pc components across participants (Sawaki et al., 2012; Tay et al., 2019) and therefore, may be a more sensitive measure than mean amplitudes. We found an interaction between the symbol position and trial type, although follow-up comparisons did not reveal any difference in area. One potential reason for this finding is the Exemplar Match trials being larger when targets appeared in the random location, compared to the patterned location. If covert attention initially was allocated to visual patterns (i.e., prior to the task-relevant trials), then attentional selection to task-relevant items should have required little to no shifts in covert attention, reflected in a smaller N2pc component, when targets appeared in the already attended location. On the other hand, a shift in covert attention is required if attention must be allocated to the opposing location, which may reflect larger N2pc components when targets appeared in the random location.

To further investigate the neural dynamics with covert attention in the exploratory 150–300 ms time window, we investigated whether scalp responses could be distinguished based on where the visual patterns occurred. For Exemplar Match trials, decoding accuracy was well-above chance across the whole 150–300 ms time window. The results are consistent with when the N2pc components occurred, suggesting that attention was directed to the location containing visual patterns. For Foil trials, above-chance performance was only during the 212–262 ms time window. We note, however, that Foil effects marked by N2pc components generally are smaller compared to Target Present trials (e.g., Wu et al., 2013, 2015, 2018), as observed in this study using mean amplitude and signed negative areas. Therefore, we expected to see a weaker (but still significant) decoding performance at a smaller time window. However, the overall observation that neural responses were distinguishable based on symbol position remains consistent.

Both jackknife and fractional area latency analyses provided converging evidence that attention is allocated faster to items when preceded by a visual pattern, compared to no visual pattern. Although both results showed this effect for Foil trials, only the jackknife analyses revealed shorter latencies with Exemplar Match trials in favor of the patterned location. Because different thresholds were used, the degrees of freedom were less for fractional area latency than jackknife, suggesting that the jackknife analyses revealed more robust effects. However, the interaction found with fractional area latency analyses, supports the conclusion that foil effects occurred faster in the patterned location than in the random location. Latencies also were shorter for Exemplar Match, compared to Foil trials, consistent with the results from other measures, showing that task-relevant activation for target representations was faster than task-irrelevant activation of categorically related non-targets.

It is likely that the neural effect during the broad time window reflects the N2pc component, and prior studies support this idea. For example, warning signals prior to a target can shorten the latency onset of the N2pc (Seibold & Rolke, 2014), as well as using fear-inducing stimuli (Eimer & Kiss, 2007; Weymar et al., 2013). Foster et al. (2020) showed that spatial cues preceding targets shift the N2pc component 20 ms faster when the cues provide information about task-relevant locations, compared to cues absent of that information. Jenkins, Grubert, & Eimer (2018) demonstrated a similar finding with target features (50 ms) and arrow cues (100 ms), as well as Robitaille & Joliocoeur (2006) with color cues. Interestingly, Kiss, Velzen, & Eimer (2008) found no difference in N2pc components regardless of whether the cues preceding the target were informative or not, suggesting that perhaps a separate early component related to attentional shifts may exist that is distinct from the N2pc. Perhaps in the present study, the patterned symbols prepared participants to select targets in that location, similar to that of attentional cues. Unlike prior work, however, the statistical patterns in the present study were irrelevant to the task, as opposed to attention cues that guide search explicitly (e.g., Woodman et al., 2009), and were only presented in one location, as opposed to being randomly occurring across trials. Despite these differences, the similarities in the early ERP components between prior studies with explicit cues and the present study with implicit statistical patterns provide interesting information on how early covert attention impacts search. More research is required to replicate these onset differences to determine how visual patterns may facilitate search, even when task-irrelevant (e.g., perhaps via accelerating visual processing, Rolke et al., 2016; Thomaschke et al., 2016).

Our neural findings align with our overall prediction that visual patterns shift attention. The exact time window when we found the effect was earlier than predicted, but is a reasonable finding given that the patterns in our study were presented prior to the target and foils. There are two possible reasons for the neural effect that we observed: One possibility is that covert attention shifted sooner in the patterned location compared to the random location (e.g., Eimer & Kiss, 2007; Grubert & Eimer, 2015), and the other possibility is that participants suppressed the random sequences prior to target and foil selection (e.g., Sawaki & Luck, 2021). The first possibility would support the idea that attention was covertly shifted to the pattern prior to the onset of the task-relevant items, and when the items appeared, attention to patterned locations was facilitated, as supported by faster latency and smaller magnitude of the N2pc. The second possibility would support the idea of suppressing the random sequences of symbols. Prior studies have noted this type of distractor suppression with the Pd (distractor positivity) component, which is a significant positive deflection prior to the N2pc (Sawaki & Luck, 2021). While the Pd has been observed for salient singleton distractors, it is interesting to consider a potentially similar effect for temporally grouped symbols. Indeed, the positive polarities for both targets and foils in the random location before the standard time window are characteristics of the Pd component. While both possibilities (selecting patterned locations and ignoring random locations) may not be mutually exclusive, selecting patterned locations aligns with prior statistical learning studies where attention is prioritized to patterned information. In addition, potential Pd components were based purely on visual inspection of a narrow time window, which may simply be noise.

There are some notable limitations to our study. Ultimately, we were unable to replicate the previously reported behavioral search advantage for targets appearing in patterned locations (Zhao et al., 2013). In addition, the neural effects did not seem to have downstream effects on later processing and behavioral responses. One critical difference between our task and the task from Zhao et al. (2013) is that the present task included only a 2-item search array, whereas the original paradigm included a 4-item search array. The average accuracy across all conditions was approximately 95%, suggesting that there were ceiling effects in our task, potentially due to using only a 2-item search array, rather than a more difficult task, such as a 4-item search array. We also designed our paradigm to better match prior N2pc studies (e.g., Wu et al., 2015), where targets and foils are only flashed on a computer screen for 200 ms, instead of remaining longer on the screen, as in the behavioral studies. There were no Target Absent trials in Zhao et al. (2013), and in that study, participants were instructed to respond by indicating which direction the target was pointing in the search array, whereas the current search task required participants to respond based on whether the target was present or absent in a given trial. These differences in task instructions may have made Zhao’s task more difficult compared to ours. Indeed, accuracy in our study was very high, and the reaction times were half of those from Zhao et al. (2013). Unpublished pilot studies by Zhao and colleagues that included 2-item search arrays also did not yield significant behavioral effects. Therefore, future research should investigate these neural and behavioral effects with a larger search array, which also aligns better with the natural search environment with more distractors. Future studies also could make the task more difficult by reducing perceptual differences among task-relevant and distractor objects (e.g., grayscale objects).

Second, the discrepancy between the neural effects and null behavioral results leads to intriguing questions related to potential search mechanisms in this study. N2pc effects often align with behavioral effects (e.g., Olivers et al., 2011). However, recent studies have revealed interesting cases where N2pc and behavioral results differ. For example, visual search studies have used the dot-probe task to measure attentional bias to threatening images (see Bar-Haim et al., 2007 for review) and include N2pc measures and manual responses. Several studies have consistently demonstrated significant N2pc components to task-irrelevant images without behavioral effects (Kappenman et al., 2014, 2015; Kiss et al., 2013). Some of our own N2pc studies using a similar two-item array paradigm to the present study have revealed significant N2pc effects but null results for reaction times (e.g., Wu et al., 2018). One explanation is that the N2pc component marks a specific neural process that activates early in the visual search process (see Eimer, 2014; Wolfe, 2021), whereas behavior occurs much later (around 600 ms) and results from the accumulation of several processes (e.g., visual search, decision making). Indeed, several studies have demonstrated not only a mismatch between N2pc and behavioral findings, but that there is poor reliability in reaction time as a psychometric measure for attentional bias (Kappenman et al., 2014, 2015; Schmukle, 2005; Staugaard, 2009; Waechter et al., 2014). This issue limits the interpretation of the potential relationship between covert attention and behavior, even if there were behavioral effects. Due to its high temporal resolution and continuous measurement, ERP analyses may be more suitable for understanding specific mechanisms compared to reaction times. In this study, perhaps the N2pc component could be a more sensitive marker of certain aspects of attentional shifts to task-irrelevant patterns compared to behavioral performance. The ERP effects drive our overall conclusion that attention to previously presented, task-irrelevant patterns is allocated early in the visual search process. More research is needed to replicate our neural findings and investigate how they may relate to behavioral effects using a more challenging task and reliable psychometric measures.

Third, in the end, we were primarily interested in the neural effects for targets and foils. Therefore, the analyses were time-locked to the search phase, as opposed to the pattern phase. The pattern phase also consisted of varying numbers of symbols (3, 6, or 9 symbols), restricting our analyses to the search phase. Therefore, a baseline correction was applied during when the runic symbols appeared, which potentially underestimated the effects observed for the early time window. If overt attention or strategic eye-movement occurred, these trials were removed after applying the artifact rejection criteria. Our analyses only included correct trials during which no or minimal overt attention occurred. Although doing so resulted in fewer useable trials, our artifact rejection criteria were consistent with other similar EEG studies (e.g., Nako et al., 2014; Wu et al., 2013, 2016, 2018). Future studies should examine the neural effects during the stream of symbols by maintaining a fixed number of items displayed during patterns, and shifting the analysis window earlier (i.e., starting from before symbols appearing to before search items appearing) to maximize the likelihood of observing the effects of implicit patterns on search outcomes. In addition, quantifying the neural effects for the pattern or random symbols also can reveal information about exactly how quick observers are at recognizing the patterns and could provide information about implicit pattern learning during a search task.

Fourth, although the number of trials between target present and foils was evenly distributed, the frequency between the appearance of specific targets and foils was different. Only one target image (e.g., carrots) appeared during Target Present trials, whereas 15 possible foils (e.g., every food item except for the carrots) appeared across the Foil trials. While this difference in frequency may have potentially contributed to some effects unaccounted for (e.g., expectations of target appearance), the main goal of the present study was to compare Target Present trials between the symbol positions (pattern vs. random) separately from Foil trials between the symbol positions. Given that the target and foils appeared evenly between the pattern and random locations, any confounding effects would most likely have occurred in both locations.

Fifth, because of plans to conduct the same study with children, we used child-friendly food and toy stimuli in this study with adults. However, if we had included a more familiar category (e.g., man-made objects) instead of toys, perhaps we would have had stronger neural and behavioral effects (Olivers, 2011; Wu et al., 2017). Future studies could investigate how the level of familiarity with the objects and categories interact with visual patterns to influence search efficiency (Ferguson et al., 2021).

In sum, our ERP findings combined with the behavioral findings from the present study and Zhao et al. (2013) suggest that visual patterns may facilitate covert attention, which may have independent effects on behavioral responses depending on task requirements. These effects were most pronounced when items appeared in the same location as the visual patterns. Overall, these results provide information on the benefits and costs of using knowledge and current input during visual search. Future studies on how prior knowledge and current input interact can lead to a better understanding of how learners find relevant information in the real world.

## Data Availability

The datasets used and/or analyzed for the present study are available from the corresponding author upon request.

## Abbreviations

EEG: Electroencephalography
ERP: Event-related potentials
IRB: Institutional Review Board
N2pc: Negative (n) ERP emerging 200 ms (2) after stimulus onset in the posterior (p) region contralateral (c) to the hemifield with the target
